# Growth-dependent activity of the cold shock *cspA* promoter + 5′ UTR and production of the protein CspA in *Staphylococcus aureus* Newman

**DOI:** 10.1186/s13104-017-2557-1

**Published:** 2017-06-27

**Authors:** Chandana K. Uppalapati, Kimberley D. Gutierrez, Gina Buss-Valley, Sam Katzif

**Affiliations:** 1 Department of Microbiology and Immunology, Midwestern University, Glendale, AZ 85308 USA; 2 Department of Immunology, University of Washington, Seattle, WA 98109 USA

**Keywords:** *Staphylococcus aureus*, Cold shock, *cspA* regulation, CspA protein, *xylE* activity, Western blot, Total protein normalization

## Abstract

**Background:**

Research involving the cold shock gene *cspA* of the medically important bacterium *Staphylococcus aureus* is steadily increasing as the relationships between the activity of this gene at 37 °C and a spectrum of virulence factors (e.g., biofilm formation, capsule production) as well as stress-related genes (e.g., alkaline shock protein, *asp*-*23* and the alternative sigma factor, *sigB*) are distinguished. Fundamental to each of these discoveries is defining the regulation of *cspA* and the production of its protein product CspA.

**Results:**

In this paper, primer extension analysis was used to identify a transcriptional start point at 112 bp upstream of the initiation codon of the *cspA* coding sequence from *S. aureus* Newman RNA collected at 37 °C. Based on the location of the putative −10 and −35 sites as well as putative cold shock protein binding sites, a 192 bp sequence containing an 80 bp promoter + a 112 bp 5′ UTR was generated by polymerase chain reaction. The activity of this 192 bp sequence was confirmed in a pLL38 promoter::*xylE* reporter gene construct. In addition, Western blots were used to confirm the production of CspA at 37 °C and demonstrated that production of the protein was not constitutive but showed growth-dependent production with a significant increase at the 6 h time point.

**Conclusions:**

The results presented identify another regulatory region for the cold shock gene *cspA* of *S. aureus* and show growth-dependent activity of both this *cspA* regulatory sequence, presented as a 192 bp sequence of promoter + 5′ UTR and the production of the CspA protein at 37 °C. The presence of two active transcription start points, a −112 bp sequence defined in this work and a second previously defined at −514 bp upstream of the *cspA* initiation codon, suggests the possibility of interactions between these two regions in the regulation of *cspA*. The growth-dependent production of the cold shock protein CspA supports the availability of this protein to be a modulator of virulence and stress factor genes at 37 °C.

## Background

*Staphylococcus aureus* is a pathogen that effects populations of companion and food animals as well as humans. The treatment options for this bacterium have become more complicated due to both community-acquired and hospital acquired MRSA, methicillin resistant *S. aureus* strains. These multidrug-resistant strains can be identified in each of these populations with the potential of anthropozoonosis [1]. As the availability of effective antibiotic treatment becomes limited, basic molecular research has become a source of identifying targets for the development of antimicrobial agents. A key component for the future treatment of bacterial diseases is the identification and disruption of the regulatory molecules responsible for modulating the genes coding for these virulence factors [2]. One such regulatory molecule and potential target for disruption in *S. aureus* is the cold shock protein, CspA.

The cold shock gene *cspA* and protein of *S. aureus* have a sequence and structure similar to that of both *Escherichia coli cspA* and *Bacillus subtilis cspB* cold shock genes and protein products [3–8]. This gene was not initially recognized in cold shock induction experiments, but identified in testing the susceptibility of *S. aureus* 8325-4 *Tn*551 insertional mutants to the antimicrobial peptide CG117-136 at 37 °C [9, 10]. The loss of *cspA* function resulted in an *S. aureus* strain with decreased susceptibility to this 20-mer peptide. Because the *S. aureus* 8325-4 strain carries a regulator of sigma B, *rsbU* mutation, the *Tn*551 insertional mutation was transduced into a *rsbU* repaired strain, *S. aureus* SH1000 [11]. The susceptibility remained but a new phenotype of carotenoid pigment reduction was identified. This pigment reduction was confirmed by a *cspA*::kanamycin cassette knockout and *trans* complementation in a highly pigmented strain of *S. aureus* COL. This was an indication that the cold shock protein CspA may have specific regulatory functions and was the first cold shock protein in *S. aureus* to be linked to virulence factors [12].

Subsequent RT-qPCR, real time-quantitative polymerase chain reaction, experiments in a *cspA* mutant of *S. aureus* COL resulted in a decrease in transcripts from *crtN*, one gene in the staphyloxanthin, a carotenoid pigment, operon, *sigB*, the virulence and stress alternative sigma factor B and *asp*-*23* (a SigB-regulated gene encoding the alkaline shock protein) [13–16]. These results were not due to the degradation of mRNA but to regulation of each gene by the cold shock protein CspA [12].

Since these initial findings, researchers have identified other distinct regulatory links among the presence of the *cspA* gene, its protein product, and other virulence factors of *S. aureus*. Among these are the modulation of a major regulator of virulence, the staphylococcal accessory regulator, *sarA*, the production of capsule in a nutrient dependent fashion, the *cap* operon, and the production of biofilm [17–19]. In relation to its regulation of *sarA*, *cspA* can now been found in the literature as the modulator of *sarA*, *msaB*, and has been reported as part of a four gene operon, *msaABCR* [20]. As part of the investigation into the regulation of the component genes of the *msaABCR* operon in *S. aureus* USA300_LAC, an identified 324 bp putative promoter for *msaB* (*cspA*) was reported as inactive and the primary promoter for the regulation of *msaB* (*cspA*) was identified as that for the *msaA* gene with a transcriptional start point (TSP) 514 bp upstream of the *msaB* (*cspA*) coding sequence [20]. In this current work, we propose a second regulatory region consisting of a 192 bp sequence and composed of a promoter + 5′ untranslated region (UTR) with a TSP −112 bp immediately upstream of the coding sequence for *cspA*. The growth-dependent activity of this sequence demonstrated by a *cspA* promoter + 5′ UTR::*xylE* reporter gene construct in *S. aureus* Newman [21] and the growth-dependent production of the protein CspA are presented.

## Methods

### Bacterial strains and plasmids

The bacteria and plasmids used or constructed for the work completed in this paper are listed in Table 1. For the construction and selection of plasmids as well as the overexpression of the cold shock protein CspA using *E. coli*, all *E. coli* strains were grown in Luria–Bertani, (LB) broth and on LB agar. All *S. aureus* strains were grown in tryptic soy broth (TSB) and on tryptic soy agar (TSA). For the broth cultures used in the XylE assays, *S. aureus* strains were grown in TSB. When required the antibiotics added to cultures were: for *S. aureus*, 20 μg/mL chloramphenicol, 25 μg/mL kanamycin, 3 μg/mL tetracycline; for *E. coli*, 50 μg/mL ampicillin, 50 μg/mL kanamycin, 25 μg/mL chloramphenicol, 50 μg/mL spectinomycin.

**Table 1:** Bacterial strains and plasmids

*Escherichia coli*		
DH5α	F^−^ Φ80 *lacZ *ΔM15Δ(*lacZA*-*argF*) U169 *deoR recA1 endA1 hsdR17* (r^−^_K_ m^−^_K_) *phoA supE44* λ^−^ *thi*-*1 gyrA96 relA1*	Invitrogen™
BL21(DE3)pLysS	F^−^ *ompT hsdS*_*B*_(r_B_^−^,m_B_^−^) *gal dcm* (DE3) pLysS (Cam^R^)	Invitrogen™
SKEPrtEx	*E. coli* BL21(DE3)pLysS with plasmid pSKPrtExA	This study
*Staphylococcus aureus*		
RN4220	*res* ^−^ strain	[23]
SKC31	A Km cassette deletion–insertion of the *cspA* coding region in *S. aureus* COL, Tet^R^	[10]
Newman	Clinical isolate, strong positive coagulase	[21]
SKN23	A Km cassette deletion–insertion of the *cspA* coding region in *S. aureus* Newman	This study
Plasmids		
pCR2.1	TA cloning vector	Invitrogen™
pSKCPriEx	pCR2.1 with 238 bp insert of *cspA* coding region, promoter + 5′ UTR	This study
pLL38	*E. coli*-staphylococcal shuttle vector that contains promoterless *xylE* as a reporter gene for promoter-gene fusion; gram-positive RBS (GGAGG)	[29]
pSK51	pLL38 containing a putative sequence of −35 and −10 sites plus P3, −53 bp putative TSP of *cspA,* ligated to a *xylE* reporter gene	This study
pSK88	pLL38 containing a putative sequence of −5 and −10 sites plus P2, −88 bp putative TSP of *cspA* ligated, to a *xylE* reporter gene	This study
pSK96	pLL38 containing a putative 96 bp sequence of the *cspA* promoter: upstream protein binding sites, −35 and −10 sites ending at P1, −112 bp putative TSP of *cspA* ligated to a *xylE* reporter gene	This study
pSK192	pLL38 containing the sequence of pSK96 + 5′ UTR of *cspA*	This study
pSK324	pLL38 containing *msaB* promoter insert from pMOE 482; 5′ *Pst*I and 3′ *Eco*RI sites	[20]; This study
pRHB152	pRHB153 with six His-tag replacement of GST-tag for protein overexpression	[25]
pSKPrtExpA	pRHB152 with six His-tag 200 bp coding region for CspA overexpression	This study

### Transduction to produce the *cspA* mutant of *S. aureus* Newman

Phage 80α propagated on *S. aureus* COL strain SKC31 [10] was used to transduce a kanamycin (Km) cassette deletion–insertion of the *cspA* coding region to *S. aureus* Newman and generate strain SKN23 (CY. Lee, personal communication). In brief, a 3 mL overnight culture of *S. aureus* Newman grown at 37 °C was diluted 1:100 in 10 mL of TSB and grown at 37 °C for 2 h. The cells were centrifuged and suspended in 0.2 mL of TSB. After the addition of 25 μL of 10 mg/mL CaCl_2_ and 30 μL of phage 80α propagated on SKC31, the infected cells were incubated at room temperature for 10 min. After a 35 min static incubation of the infected cells at 30 °C, 5 mL of TSB was added, the cells centrifuged, and the pellet suspended in 5 mL of TSB. The infected cells were incubated at 37 °C for 1.5 h. The cell pellet was suspended in 0.2 mL of TSB and plated onto three TSA plates containing 25 µg/mL of kanamycin and incubated at 37 °C for selection of *cspA*::Km deletion–insertion mutants. The replacement of the *cspA* coding sequence by the Km cassette in a selected *S. aureus* Newman colony SKN23 was confirmed by PCR, polymerase chain reaction.

### Primer extension analysis of the 5′ UTR of *cspA*

Primer extension was carried out with RNA collected from *S. aureus* Newman cells (QIAGEN RNeasy kit) grown at 37 °C, harvested at 0.3–0.4 at OD_600_, and diluted to a CFU/mL of 1 × 10^8^. From the total RNA isolated from these cells, a 20 μg aliquot was first annealed to the 5′-^32^P-labeled primer R-CPriEx and then combined with AMV reverse transcriptase (Promega) to generate complementary DNA (cDNA) primer extension fragments (Table 2). The template for a parallel GATC sequencing reaction was provided by pSKCPEx that contains a 238 bp putative *cspA* promoter region and coding sequence. The 238 bp insert is a PCR, (ARKTIK Thermocycler, Thermo Fisher) product of the primers F-CspA238 and R-CspA238 combined with genomic DNA (gDNA) from *S. aureus* Newman (Qiagen DNeasy Blood and Tissue kit) (Table 2). The 238 bp product was gel purified (QIAGEN Qiaquick Gel Extraction kit), ligated into a pCR2.1 vector (Invitrogen™) and transformed into *E. coli* DH5α. The transformants were selected on LB agar containing 40 μg/mL X-Gal and 50 μg/mL kanamycin. The presence of a 238 bp insert in the pSKCPEx plasmid was confirmed by restriction digestion with *Eco*RI (Promega) and DNA sequencing (DNA Laboratory at Arizona State University). Using a Sequenase 2.0 sequencing kit (Affymetrix), unlabeled primer R-CPriEx annealed to 4 μg of pSKCPEx and then labeled with dATP-α-^33^P was used to create each of the GATC sequencing reactions. The labeled primer extension cDNA was loaded onto an 8% polyacrylamide gel and run simultaneously with the DNA sequencing reactions of pSKCPEx. Primer extension fragments and the GATC sequence were detected by phosphor screen imaging (GE Healthcare) and analyzed for transcriptional start points (TSP).

**Table 2:** Primers

R-CPriEx	CCATTTAACTGTACCTTGTTTCATAATCTGAAACC
F-CspA238	GTTTCATTGTTTACAAAATAATGAAGTATATT
R-CspA238	CCTTGTTTCATAATCTGAAACCTCC
F-P1/96-192	CAAAATACTGCAGTATATTATAAACTACC
F-P2/88	GTACTGCAGTTTGGTAATAACTGC
F-P3/51	GTTAAGCTGCAGATTATTCCATATTGC
R-P3′	CCTCGAATTCTAAAATTCATTCAATATGC
R-P96	GAATTCTGCTTAACTTGTATTATAGTGC
F-P324	TGCGAAGATCTGCAGGAGGATTACAAATATTTTA
R-P324	ACGTCATTGAATTCTTCAACTTCGATAAAGCC

### Construction of the *cspA* promoter region ± the 5′ UTR::*xylE* reporter plasmids

For the construction of each of the putative *cspA* promoter region or promoter region + 5′ UTR::*xylE* reporter gene plasmids (Table 1), a standard protocol was used. In brief, a set of 5′ and 3′ primers (Table 2) for each of the five pLL38 *xylE* inserts, P3 (51 bp), P2 (88 bp), *cspA* promoter (96 bp), *cspA* promoter + 5′ UTR (192 bp), and a 324 bp sequence, was combined with gDNA extracted from overnight cultures of *S. aureus* Newman for a PCR. The primer R-P3′ was paired with each of the 5′ primers for all PCR except the 96 bp *cspA* promoter which required primer R-P96. The 5′ primer for the 192 and 96 bp inserts is the same, F-P1/96-192. Each PCR fragment was gel-purified and ligated into the cloning vector pCR2.1 and transformed into *E. coli* DH5α to provide plasmids (Qiagen QIAprep Spin Miniprep Kit) to confirm the sequence of each insert. Each insert was removed by a double-digest, *Pst*I and *Eco*RI, of the pCR2.1 construct, gel purified and ligated into a similarly digested *xylE* reporter vector pLL38 [29] and transformed into *E. coli* DH5α. After confirmation of the sequence and orientation in pLL38, plasmids were first transformed into *S. aureus* RN4220 by electroporation using a Gene Pulser Xcell™ (Bio-Rad) followed by extraction and then electroporation into the target strain, *S. aureus* Newman [21–23].

### Assays for *cspA*::*xylE* reporter gene constructs

The protocol published by Torres et al. [24] was adapted for these assays. In brief, 25 mL of TSB with no antibiotics were inoculated (initial OD_600_ between 0.074 and 0.080) from an overnight culture of a *cspA*::*xylE* reporter construct. The culture was grown at 37 °C. Optical density (OD_600_) was measured (0–6 h) and samples were taken after 1 h (8 mL), 3 h (4 mL) and 6 h (0.5 mL) of growth. Times are comparable to pre-logarithmic, mid-logarithmic and post-logarithmic growth. Each sample was centrifuged and the pellet suspended in potassium phosphate buffer (20 mM potassium phosphate buffer pH 7.5) and centrifuged. The cell pellets were immediately stored at −80 °C and the XylE assay performed within 24 h after sample collection. The protein from each pellet was extracted with 150 μL of lysis buffer (100 mM potassium phosphate buffer pH 8.0; 10% (v/v) acetone; 25 μg/mL lysostaphin), incubated at 37 °C for 20 min and placed on ice for 5 min before a final centrifugation.

For each assay, a 20 μL volume of the supernatant from each timed sample was combined with 200 μL of the reaction substrate (22.5 mL ddH_2_O; 2.5 mL 1 M potassium phosphate buffer pH 8.0 and 2.5 μL 0.2 M Catechol) in a 96 flat-bottom well, microtiter plate for each assay (Greiner Bio-One). The specific velocity of 2-hydroxymuconate semialdehyde production was determined on a BioTek Cytation™3 Multi-Mode reader (BioTek Instruments, Inc). Readings of this kinetic reaction were taken at 375 nm for 30 min at 30 °C. The change in concentration of 2-hydroxymuconate semialdehyde was calculated in nmol/min. A bicinchoninic acid (BCA) protein assay (Pierce Protein Research Products) endpoint reaction was used to determine the protein concentration of each sample. Each protein sample (20 μL total volume, 10 μL lysis supernatant + 10 μL XylE lysis buffer) was combined with 200 μL of bicinchoninic acid reaction substrate for this assay. The change in concentration of 2-hydroxymuconate semialdehyde was divided by mg protein to generate units of XylE specific activity (nmol/min/mg protein).

### CspA overexpression, purification, and antibody production

For the overexpression of CspA using a His-tagged construct, PCR with the primers F-CPrtnNdeI and R-CPrtnXhoI was used to produce the *cspA* coding region from *S. aureus* Newman gDNA, The 201 bp PCR product was gel purified, ligated into pCR2.1, and transformed into *E. coli* DH5α. After selection on LB agar, putative colonies were grown overnight in LB broth at 37 °C. Plasmids were extracted, digested with *Eco*RI to confirm the presence of an insert, and sequenced to confirm a correct insert. After sequence confirmation, the plasmids were digested with *Nde*I and *Xho*I (Promega) and the inserts ligated into an identically restriction digested and gel-purified overexpression vector pRHB152. The construction of the pRHB152 plasmid is identical to the pRHB153 plasmid with the replacement of the GST-tag with a six His-tag sequence [25] (Hernandez, personal communication). The overexpression construct pCProtEx was transformed into *E. coli* BL21(DE3)pLysS competent cells. The transformants were selected by plating on LB agar plates at 37 °C. The presence of a correct *cspA* coding sequence, and sequence alignment with the His-tag for read-through transcription were confirmed in selected isolated colonies. For overexpression of CspA, cultures were grown in LB broth with selection to 0.600 at OD_600_ and protein production was induced by adding 1 mM IPTG. After 3 h, the induced cultures were harvested by centrifugation. Each cell pellet was washed twice with PBS pH 7.3 and centrifuged. The cell pellet for each sample was flash frozen with liquid nitrogen before storing at −80 °C. For purification, 10 mg of frozen cells were suspended in buffer (50 mM Tris–HCl pH 8.0, 50 mM NaCl, 0.5 μg/mL leupeptin, 2 mM PMSF, and 5 μg/mL DNase I) and lysed by sonication on ice (Fisher Scientific™ Sonic Dismembrator Model 500). The crude cell lysate was centrifuged for protein extract and the His-tagged CspA protein purified from the soluble protein using Nickel bound to Chelating Sepharose Fast Flow beads (GE Healthcare) at 4 °C.

### Immunoaffinity purification of the rabbit-CspA antibody for Western blots

Polyclonal rabbit antibodies raised against a purified CspA protein [26] were produced by the Proteintech Group Inc. To reduce the amount of secondary binding observed in preliminary Western blots, the rabbit antiserum against purified CspA was further purified following an Affinity-Purification of Antibodies via Protein-Coupled Sepharose beads protocol (Proteintech). In brief, purified CspA was coupled to CNBr-activated Sepharose™ 4B beads (GE Healthcare) in the presence of a coupling buffer (100 mM NaHCO_3_, 500 mM NaCl pH 8.3) and incubated for 2 h at room temperature. The protein coupled beads were then washed three times with PBS followed by incubation with rabbit anti-CspA antiserum overnight at 4 °C. The protein-bead-antiserum suspension was loaded onto a 10 mL column, and washed with 10 mL PBS pH 7.4, and then 10 mL of 150 mM NaCl–HCl pH 5.0. The protein was eluted from the column with 6, 0.9 mL fractions of 150 mM NaCl–HCl pH 2.75 and the eluent was immediately neutralized with 0.1 mL of saturated PBS pH 7.5.

### Western blot analysis and quantitation of CspA production

CspA protein production at different growth phases of *S. aureus* Newman and SKN23, a *cspA*::kanamycin cassette mutant, was initiated by growing the two strains overnight in TSB at 37 °C. The overnight cultures were normalized at an OD_600_ between 0.075 and 0.08 in 125 mL TSB and aliquots were harvested at 1, 3, and 6 h time points post dilution. Each aliquot was centrifuged, washed with 20 mM potassium phosphate buffer pH 7.5 and the pellet frozen at −80 °C until extraction. For the extraction of total cellular protein, each cell pellet was suspended in 500 μL of buffer (25 mM Tris–HCl, 100 mM NaCl, 5 mM EDTA pH 8.0 buffer, 0.5 μg/mL leupeptin, 2 mM PMSF, and 5 μg/mL DNase I). Then 50 μg/mL lysostaphin was added and the cells incubated at 37 °C for 30 min followed by ice incubation for 1 h. Cells were subjected to sonication on ice. Whole cell lysates were centrifuged at 4 °C (Sorval ST16R, Thermo Scientific), to collect supernatants that were centrifuged again at 4 °C. The clear supernatants were collected and analyzed for total protein concentrations using BCA. Equal concentrations of 30 μg of total protein for each sample were heat denatured at 95 °C for 5 min and quick chilled before being loaded onto a 15% SDS-polyacrylamide gel. The electrophoresis resolved proteins were electroblotted onto 0.2 micron PVDF membrane (Bio-Rad Mini Protean 3 gel system, Millipore Immobilon P^SQ^), air dried, and stained with Ponceau S to confirm total protein transfer. The blot was rinsed with water followed by 1% (v/v), Tris-buffered saline with Tween 20 (TBST) and blocked for 48 h with 5% (w/v) non-fat dry milk in 1% (v/v), TBST. CspA protein was detected with a rabbit-anti-CspA (1:1000 dilution) followed by goat anti-rabbit IgG horseradish peroxidase (HRP) (1:10,000 dilution) incubation. CspA bands were exposed to ECL Prime substrate (GE Amersham) and were detected using a Bio-Rad Chemi-Doc (Universal Hood III) gel imaging system. To measure the relative expression, CspA bands were quantitated using Image lab 5.2.1 software following a total protein normalization method (Bio-Rad).

### Statistical analysis

A t test was used to determine the statistical significance between samples at p ≤ 0.05. All experiments were carried out ≥3 times and all samples were measured in triplicate except for the Western blot data in which duplicate samples were loaded and run on the same or simultaneously on a separate polyacrylamide gel.

## Results and discussion

### Identification of a TSP for *cspA* in *S. aureus*

**Fig. 1:**
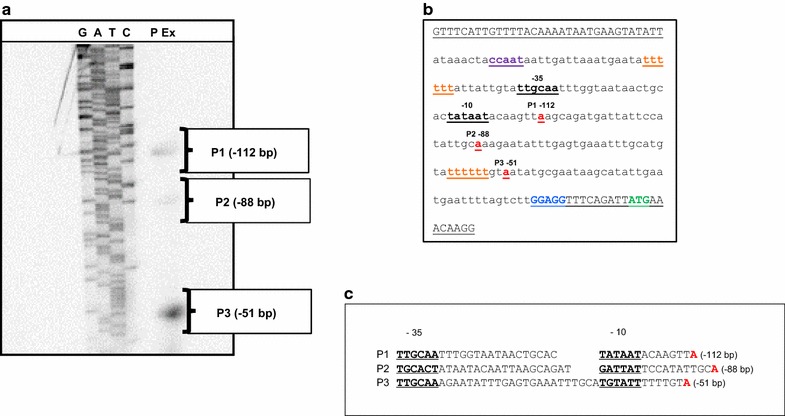
Primer extension to identify a transcription start point for the cold shock gene *cspA*. **a** The *lanes* for each of the specific nucleotide bases of the *cspA* sequence are labeled G, A, T, C. P Ex identifies the primer extension fragments. The putative transcription start points, P1 (−112 bp), P2 (−88) and P3 (−51) are labeled with *bold letters* and *numbers*. *Brackets* indicate the cDNA primer extension fragments. **b** The 238 bp sequence used to identify the locations for the P1 (−112 bp), P2 (−88) and P3 (−51) TSP. Each proposed TSP is labeled and the corresponding nucleotide is in *bold red* and *underlined*. The 5′ and 3′ primers are in *capital letters* and *underlined*. The translation start codon for *cspA* is in *bold green* and a ribosomal binding site is in *bold blue*. The putative −10 and −35 RNA polymerase binding sites are labeled and the sequence is in *bold black* and *underlined*. The proposed binding sites, CCAAT and TTTTTT, for a cold shock protein are labeled in *bold purple* and *bold orange*, respectively and *underlined*. **c** The putative promoter sequences for each of the putative TSPs. Each sequence is identified by P1, P2 and P3 on the *left*, the putative TSP in *bold red* at the 3′ end, and sequence location in *parentheses* at the *right*. The putative −35 and −10 sequences are labeled and in *bold black underlined letters*. Space has been added in the P1 and P2 sequences to align the −10 sites

Based on previous work in defining a 5′ UTR, untranslated region, upstream of a putative coding sequence in the cold shock gene *cspA* of *S. aureus* [10], a primer extension protocol was used to distinguish an actual TSP for the promoter of this gene. The primer extension analysis revealed three putative fragments (Fig. 1). These putative transcripts begin at −112 (P1), −88 (P2), and −51 (P3) base pairs upstream of the start site for translation (Fig. 1). Only one TSP had been previously reported in the cold shock genes, *cspA* of *E. coli* and *cspB* of *B. subtilis* [4, 7]. The presence of one TSP per cold shock gene changed when the production of multiple start sites was described in the primer extension analysis of the cold shock genes *cspC* and *cspP* in *Lactobacillus plantarum* [27]. The smaller primer extension fragments observed for both *cspC* and *cspP* arose from specific degradation of the mRNA and the actual transcription initiation sites of these two genes corresponded to the 5′ ends of the longest extension products [28]. This work was followed by the identification of three TSPs in both the *cspA* and *cspB* cold shock genes of *Caulobacter crescentus* [28]. To test the activity of each of these primer extension fragments, promoter-like sequences for each of these fragments were created by PCR through the production of sequences that included putative −10 and −35 RNA polymerase binding sites upstream of each potential start site (Table 2; Fig. 1) and ligating each of these to the *xylE* reporter gene of plasmid pLL38 [29]. The XylE analyses of these plasmids, pSK51 containing the P3 fragment and pSK88 with the P2 fragment, produced a range of activity (0.00–0.17 units of XylE specific activity) (Table 3). We subsequently classified both pSK51 and pSK88 constructs as inactive.

**Table 3:** XylE units of specific activity for the putative P2 and P3 promoters

	1 HR	3 HR	6 HR	Average
P2 (−88 bp)^a^	0.13	0.17	0.00	0.10
P3 (−51 bp)^a^	0.12	0.04	0.00	0.05

Units of specific activity = nmol/min/mg protein

^a^Data normalized by subtraction of promoterless vector pLL38 XylE velocity before calculation of units specific activity

The 112 bp putative P1 promoter containing a 5′ UTR was combined with an additional 80 base pairs upstream from the −112 bp putative TSP. These 80 bp contain a putative consensus TATAAT, −10 and a TTGCAA, −35 RNA polymerase binding site as well as proposed cold shock protein binding sites of a hexathymidine sequence and a pentamer CCAAT (Fig. 1) [5, 34]. This 192 bp putative promoter + 5′ UTR::*xylE* reporter gene construct pSK192 demonstrated XylE activity in *S. aureus* Newman (Fig. 3). The change in the level of units of XylE specific activity of pSK192 was not statistically significant when comparing the 1–3 h (p > 0.05) but increased significantly, ~14× at 6 h (p ≤ 0.05) (Fig. 3). In recent work, each of these fragments has been identified in relation to the endonuclease activity of RNase III [30]. The 113 bp (P1 −112 bp) fragment represents a full length 5′ UTR of the *cspA* gene. The −88 bp (P2 −88 bp) and the −53 bp (P3 −51 bp) are the products of post transcriptional modification of this 5′ UTR by RNase III to increase the stability of the *cspA* mRNA and increase the binding affinity of the *cspA* mRNA to a ribosome [30]. This results in an increased production of the protein product CspA. This work adds support to our findings of the P3 and P2 fragments as inactive and not transcriptional regulatory sequences and the P1 fragment as a 5′ UTR of a larger regulatory sequence. In our study, we have identified the promoter component to the 5′ UTR as an 80 bp sequence (Fig. 1). To determine the contribution of the 5′ UTR to the regulation of *cspA*, we constructed a putative promoter only plasmid pSK96 and tested for XylE activity at the same time points of 1, 3, and 6 h for comparison to the activity of pSK192. When compared to pSK192, the overall results for pSK96 showed a similar pattern of growth-dependent activity with significantly less XylE activity at the 3 h and 6 h time points (p ≤ 0.05) (Fig. 3), This reduction in units of XylE specific activity by pSK96 at 3 and 6 h could be related to the loss of the 5′ UTR that results in the absence of a potential regulatory protein binding site within the 5′ UTR (i.e., the hexathymidine at −54 to −59 bp) (Fig. 1) [31], a change in the secondary structure of the shortened mRNA that limits ribosomal binding, or a decrease in longevity of the mRNA. We feel it is important to note that these experiments demonstrate that the 5′ UTR is a component of regulation and the 80 bp putative promoter sequence also makes a contribution to the activity of these XylE constructs and potentially to *cspA*. The specifics of these latter contributions will need further work.

### Comparison of the structure and activity of the 192 bp XylE active sequence to a reported inactive 324 bp sequence

**Fig. 2:**
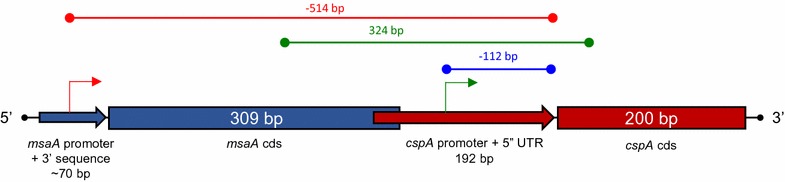
A chromosomal arrangement of regulatory sequences and two proposed *cspA* transcriptional start points in *Staphylococcus aureus*. The location and length in base pairs (bp) for the promoter + 3′ sequence (*arrow*) and coding sequence (cds) (*rectangle*) for *msaA* in *light blue* are shown in relation to the length and location of the promoter + 5′ UTR (*arrow*) and cds (*rectangle*) for *cspA* in *red*. The TSP for *cspA* identified by primer extension is identified by a *green bent arrow* and located by a 112 bp *dark blue overline* upstream of the 200 bp coding sequence for the *cspA* gene in *red*. The initial promoter for *cspA*/*msaB* identified by Sahukhal and Elasri [20] is identified by a *red bent arrow* at the 5′ end of the proposed promoter for *msaA* in *blue* and located by a 514 bp *red overline* upstream of the cds for *cspA* [20]. The *red* 192 bp promoter + 5′ UTR of *cspA* designed for the XylE assays is labeled. The location of the 324 bp regulatory sequence used for XylE assays is marked by a *green overline* [20]. The relative locations of these features are based on sequences from *S. aureus* Newman (NC_009641.1) [33]

**Fig. 3:**
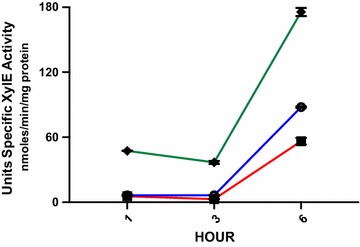
The activity of *cspA* sequence::*xylE* reporter gene constructs in *Staphylococcus aureus* Newman. Units of XylE specific activity were measured over a 6 h time period with samples collected at 1, 3, and 6 h. The values for the *cspA* promoter::*xylE* plasmid pSK96 are represented by *black-filled squares* joined by *red lines* and the *cspA* promoter + 5′ UTR plasmid pSK192 values are *open circles* linked by *blue lines*. The 324 bp sequence::*xylE* reporter construct pSK324 is represented by *black-filled diamonds* connected by *green lines*. The XylE velocity for each time point was normalized by subtraction of the velocity of the promoterless *xylE* reporter plasmid pLL38 before the units specific activity (nmol/min/mg protein) were calculated. *Double-ended error bars* represent the standard error of the mean for each time point

The 192 bp active promoter + 5′ UTR identified in this work is a distinct sequence within the previously identified inactive 324 bp *cspA*/*msaB* sequence reported by Sahukhal and Elasri [20] (Fig. 2). This 324 bp sequence contains an additional 29 bp upstream of the 5′ end of the 192 bp construct and extends 103 bp downstream of the 3′ end of the 192 bp *cspA* promoter + 5′ UTR and 31 amino acids into the coding region of the *cspA* gene (*msaB*). Interestingly both sequences extend into the 3′ end of the coding region for the proposed upstream *msaA* gene, seven amino acids for pSK192 and 20 amino acids for the 324 bp sequence (Fig. 2) [20]. Although the sequence for the 324 bp in the previous work was obtained from *S. aureus* strain USA300_LAC (NC_ 007793.1) [32], the alignment of the sequence for this chromosomal segment is 100% identical to that found in *S. aureus* strain Newman (NC_009641.1) [33]. The sequence for the 5′ UTR and the coding sequence for *S. aureus cspA* have been previously submitted as AF259960 to GenBank [10]. Since the XylE active 192 bp promoter + 5′ UTR sequence is a component of this inactive 324 bp sequence, we decided to test the activity of this 324 bp in a *xylE* reporter construct pSK324. In the work reported by Sahukhal and Elasri [20], this 324 bp linked to the *luxAB* reporter gene of pCN58 showed no activity. However in our hands this same 324 bp sequence showed activity in excess of and significantly greater (p ≤ 0.05) than both pSK96 and pSK192 at all 3 collection times (Fig. 3). The pSK324 units of XylE specific activity also demonstrated a similar growth-dependent pattern to pSK96 and pSk192 plasmids. There are possible reasons that could be related to no activity in the pCN58 construct pMOE 482 and activity in the pLL38 construct pSK324. When we generated the 324 bp insert by PCR, we used the same primer sequences [20] (Table 2), except for changes in the sequence for the restriction sites (5′ *Pst*I for *Bam*HI and 3′ *Eco*RI for *Kpn*I) for ligation to the *xylE* reporter gene in pLL38. These new restriction sites introduced a total of 10 bp changes, 6 at the 5′ *Pst*I and 4 at the 3′ *Eco*RI, These changes could effect the confirmation of the DNA or the mRNA and effect the process of transcription and/or translation. The origin of replication (*ori*) and copy number for each plasmid are different, The *ori* for pCN58 is from pT181 with a copy number of 22 and the *ori* for pLL38 is from pC194 with a copy number of 15 [34]. In this case the copy number appears to favor pCN58 in terms of expression. Another source of this measurable activity in pSK324 could be due to the kinetic protocol of XylE expression over 30 min versus a protocol of luciferase expression that requires measurement within a 10 s time frame [20]. The former protocol provides a longer time for the development of measurable end product. Resolution of this discrepancy between these results could occur through the transformation of pSK324 into *S. aureus* USA300_LAC and test for XylE activity, or the transformation of *S. aureus* Newman with pMOE 482 and test for luciferase activity. A third possibility is to use another promoter::reporter plasmid such as pGL or pXEN-1 in both *S. aureus* backgrounds [35, 36].

### Production of the CspA protein over a time course of 6 h

**Fig. 4:**
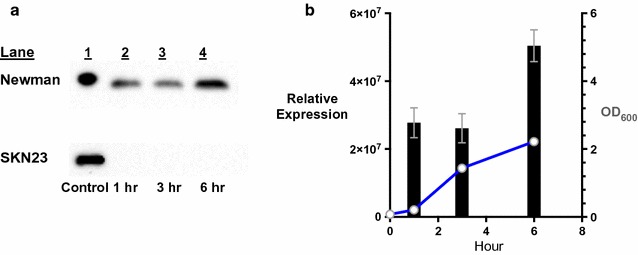
CspA protein production and growth of *Staphylococcus aureus* Newman. **a** The Western blot of CspA production by the wild type *S. aureus* Newman is the *upper band*. The *lower band* is the Western blot of a negative control *S. aureus cspA* mutant SKN23. *Lane 1* contains 35 ng of purified His-tag CspA control. In both the wild type and mutant Western blots, *Lanes 2*, *3*, and *4* contain 30 µg of total soluble protein from aliquots of cells taken at 1, 3, and 6 h. The Lane, Control, and h for each sample are labeled. **b** The relative expression values for CspA bands were calculated using Image Lab 5.2.1 software following a total protein normalization method. The relative expression of CspA for each time point, 1, 3, and 6 h, is represented by *black columns* with *double-ended bars* for the standard error of the mean (SEM) (*left* Y axis). The OD_600_ for each sample is represented by a *gray circle* and time points connected by *blue lines* with *doubled-ended error bars* for the SEM (*right* Y axis). The *double-ended error bars* are not shown if the SEM is less than the width of symbol. The OD_600_ sample for 6 h was diluted 1:2 before measurement

In using the vector pRHB152, a six His-tag CspA expression construct, pSKPrtExpA was created and used to produce a soluble form of the protein. The CspA protein was purified from the total soluble component of sonicated *E. coli* BL21(DE3)pLysS cells after 3 h of induction. The CspA protein was used to generate and to affinity purify rabbit anti-CspA antiserum. Based on the results of Western blots, over the time course of 6 h, the CspA protein is present in the wild type culture from samples taken at 1, 3, and 6 h. Using a total protein normalization method (BioRad), the calculated amount of CspA production at 6 h was significantly greater (p ≤ 0.05) than the other two timed collections (Fig. 4). These data support the production of CspA in a growth-dependent fashion and not constitutively. These results also add support to the regulation of the *msaABCR* operon by an anti-sense RNA of *msaR* in a growth phase dependent fashion proposed by Sahukhal and Elasri [20, 30]. These investigators demonstrated that the anti-sense RNA of *msaR* is present in both the pre-logarithmic and mid-logarithmic phase of growth yet absent in the post-logarithmic phase of growth. If the production of CspA seen in this study is superimposed over the production of the anti-sense RNA of *msaR*, it is possible to see a lower production of CspA at 1 h (pre-logarithmic) and 3 h (mid-logarithmic) growth in the presence of the *msaR* anti-sense RNA and the increase production of CspA at 6 h (post-logarithmic) growth in the absence of this RNA. These findings also appear to align with the production of a shorter and more active transcript of *cspA* by RNase III degradation of a longer and less active transcript through increased access to *cspA* mRNA in the absence of the anti-sense RNA of *msaR* [30]. Verification of this same data, production of the anti-sense RNA *msaR* in *S. aureus* Newman and CspA production in *S. aureus* USA300_LAC, seems highly likely based on the 100% identity in the alignment of these two sequences for the *cspA* 5′ UTR and coding sequences.

## Conclusions

In this paper, we present results that show a growth-dependent relationship between the production of the cold shock protein CspA (Western blot) and the activity of a *cspA* promoter + 5′ UTR (units of XylE specific activity) adjacent to the *cspA* coding region. CspA is produced in a growth-dependent fashion that significantly increases at the post-logarithmic time point of 6 h. This production of CspA at 37 °C demonstrates the availability of this protein to modulate virulence factors (pigment and biofilm production, antimicrobial peptide susceptibility, capsule) and the major regulatory molecule SarA. The growth phase dependent increase in the production of CspA also supports the association between the transcription of *cspA* and regulation by a proposed anti-sense RNA identified as *msaR* [20, 30]. The identification of this *cspA* promoter + 5′ UTR adds both definition and potential complexity to the regulation of the *cspA* gene. The results presented define a new *cspA* regulatory region, designed as a 192 bp sequence composed of a promoter region + a 5′ UTR upstream of the CspA translation start site. The presence of two active promoters, one initiating transcription at 112 bp upstream of the cold shock initiation codon defined in this work and the second initiating transcription from the promoter of the proposed upstream gene *msaA* [20] at 514 bp upstream of the *cspA* initiation codon, suggests the possibility of interactions in the regulation of *cspA*. We are currently pursuing the study of this interaction with recombinant chromosomal constructs that will allow us to identify the activity of and report the contribution of each of these promoters to the regulation of *cspA* transcription and CspA protein production both in vitro and in vivo.

## Abbreviations

TSB: tryptic soy broth
LB: Luria–Bertani medium
BCA: bicinchoninic acid
TSP: transcriptional start point
UTR: untranslated region
PCR: polymerase chain reaction
RT-qPCR: real time quantitative polymerase chain reaction
cDNA: complementary DNA
gDNA: genomic DNA
Km: kanamycin
Tet: tetracycline
